# Resilience and frailty among gynecological patients in oncological treatment: the chain mediating role of stigma and health literacy

**DOI:** 10.3389/fpsyt.2025.1497074

**Published:** 2025-04-02

**Authors:** Shuo Man, Xiaofang Wu, HaoWen Huang, Jinjin Yu, Ling Xia

**Affiliations:** ^1^ Obstetrics and Gynecology Department, Affiliated Hospital of Jiangnan University, Wuxi, Jiangsu, China; ^2^ Jiangnan University Wuxi Medical College, Wuxi, Jiangsu, China

**Keywords:** gynecologic oncology, frailty, resilience, stigma, health literacy, mediation effect

## Abstract

**Background:**

Frailty poses a significant threat to the health status among gynecological patients in oncological treatment. Previous studies have shown that resilience, stigma, and health literacy are associated with frailty. However, their effects and potential relationship with frailty among gynecological patients in oncological treatment have not been fully studied.

**Objective:**

The study aimed to explore the mediation role of stigma and health literacy between resilience and frailty among gynecological patients in oncological treatment.

**Methods:**

A total of 202 gynecological patients in oncological treatment in three gynecological wards of a tertiary hospital in Wuxi from March 2024 to May 2024 were selected for the study using a cross-sectional research method. A convenience sampling method was used. Information was collected on demographic characteristics, resilience, stigma, health literacy, and frailty. The PROCESS macro program was used to explore the mediating role of stigma and health literacy in the relationship between resilience and frailty.

**Results:**

The prevalence of frailty among gynecological patients in oncological treatment was 27.2%. The mediation effect results demonstrated that resilience was not a direct predictor of frailty (β = -0.010, 95%CI: -0.084 ~0.065) but indirectly predicted frailty through health literacy (β = -0.100, 95%CI: -0.155 ~ -0.053) and stigma-health literacy (β = -0.056, 95%CI: -0.091 ~ -0.029). However, no significant mediating effect of stigma alone was found (β = -0.038, 95%CI: -0.085 ~ 0.005).

**Conclusions:**

The findings reveal the impact and potential relationship of resilience and frailty among gynecological patients in oncological treatment in patients with gynecologic oncology. Health literacy mediated the effect between resilience and frailty. Stigma and health literacy were chain mediators in the link between resilience and frailty. Healthcare professionals should pay timely attention to the psychological and mental status of gynecological patients in oncological treatment patients with gynecologic oncology and take measures to improve health literacy.

## Introduction

Gynecologic tumors refer to neoplasms that occur in the female reproductive organs, which generally include malignant tumors such as cervical cancer, ovarian cancer, endometrial cancer, and vulvar cancer, as well as other benign tumors like uterine fibroids and ovarian cysts (1). It is estimated that the annual death cases of gynecologic tumors in China for the year 2022 are approximately 101,800 (2). Gynecologic tumors lead to impaired reproductive function, psychological abnormalities, difficulties in sexual life, and tremendous social and cultural pressure, which undoubtedly bring harm to patients.

Frailty is a biological condition resulting from a cumulative decline in the functioning of multiple physiological systems (3). It is essentially characterized by increased sensitivity to stress and decreased functional reserve (3). Specifically, frailty can manifest as physical, psychological, and social frailty (4), which increases an individual’s susceptibility to adverse outcomes (5). Frailty is a dynamic process influenced by various factors, including the external environment and intrinsic characteristics, such as an individual’s psychological and behavioral skills (6). In addition, treatment modalities, such as surgery, may further exacerbate the patient’s frailty and increase the risk of postoperative adverse health outcomes (7). Several studies have investigated gynecologic cancers, reporting rates ranging from 6.0% to 60.0% (8–10). Studies have shown that unexplained weight loss in major gynecological surgeries is significantly correlated with increased rates of postoperative complications and that unexplained weight loss is considered part of frailty (11). Most notably, the prevalence of physical frailty is almost twice as high in women (9.6%) as in men (5.2%) (12). The psychosocial impact of frailty is particularly severe for women, making them more prone to problems such as depression, anxiety, and loneliness (13). Therefore, there is an urgent need to prevent and manage frailty among gynecological patients in oncological treatment.

Resilience is the process of taking a positive approach to responding effectively and working to maintain equilibrium in the face of stress, threat, and adversity (14). Adversity is a situation or event generally recognized as depleting or exceeding an individual’s resources in a given context and may interfere with the individual’s normal functioning (15). According to the frailty fulcrum’ model (16), resilience may be a key contributor to frailty. Related studies have shown that high resilience is associated with good physical and mental health (17), social belonging (18), and health behaviors in individuals (19), which overlap with factors that reduce frailty. Some evidence suggests that high levels of resilience are an essential factor in improving quality of life and health outcomes (20–22). Previous studies have reported that patients with better resilience are more likely to choose an upbeat coping style to deal with stressors, which is a negative predictor of frailty (23, 24). Accordingly, we proposed hypothesis 1 (H1): resilience directly predicts frailty.

In addition, resilience is malleable, and health literacy is considered an important asset in building resilience (25). Health literacy refers to an individual’s ability to access, understand, and use health information to prevent disease and promote health (26). Previous studies have found that multilevel health literacy interventions significantly increase individual resilience. Such interventions include healthcare access and utilization (navigation and pathways), healthcare professionals-patient interactions (communication and knowledge transfer), and self-management (motivation and self-efficacy) (27). Furthermore, providing health education materials that are easy to understand and operate helps individuals acquire knowledge and skills related to health, which is vital in enhancing their ability to mobilize their resilience in the face of adversity (28). Several studies have shown that health literacy is significantly and positively associated with resilience (29, 30).

The integrated health literacy model explains the antecedents and consequences of health literacy. It proposes that individual, situational, social, and environmental determinants influence health literacy and, ultimately, frailty (31). Previous studies have shown that nursing interventions aimed at improving health literacy can effectively ameliorate frailty in elderly community-dwelling individuals (32). Individuals with better health literacy are less likely to suffer from physical and mental frailty, and most of the current research on health literacy and frailty has supported this negative association. For example, in a cross-sectional survey of hypertensive and diabetic patients in Sichuan Province, China, health literacy was negatively associated with frailty through the mediating variable of social support (33). A study of Chinese community-dwelling older adults found that health literacy was an independent negative predictor of frailty after adjusting for education (34). Furthermore, a survey among community-dwelling older adults in Japan also revealed a negative association between health literacy and frailty (35). More importantly, the researchers reported that health literacy mediated the relationship between resilience and health outcomes (36). Accordingly, we made hypothesis 2 (H2): health literacy mediates the effect between resilience and frailty.

Stigma is an internal experience of shame due to the illness and is a psychological stress response (37). According to Pérez-Flores, N. J., stigma affects an individual’s behavior in seeking health information and resources. Individuals may avoid seeking health help due to fear of being labeled or experiencing social exclusion, which limits their access to and processing of health information and affects health literacy (38). Intervention programs implemented under the health stigma and discrimination framework have shown that stigma affects an individual’s health literacy. Stigmatization may lead people to avoid regular medical check-ups or follow treatment plans because they are afraid of exposing their health problems and thus experience more discrimination (39). Previous studies have shown that diabetes-related stigma is negatively associated with health literacy (40).

The biopsychosocial model emphasizes the interaction of physiological, psychological, and social factors that significantly impact health status (41). This model provides a strong rationale for identifying and intervening on variables that can improve health outcomes. There is evidence that psychosocial factors (e.g., stigma) promote the progression of frailty (42). Stigma is a complex emotion produced by social and psychological factors. The study found that in China, women experienced more severe stigmatization (43, 44). Gynecologic oncology-related stigma refers to the feeling of stigmatization among gynecological patients in oncological treatment due to their experience of being discriminated against and mistreated by society because of their diseases. Gynecologic oncology-related stigma is a significant barrier to gynecologic care, as it can impede social interactions and daily activities and may trigger psychological problems such as anxiety and depression, which can increase the risk of frailty (45, 46). Shafig argues that frailty has negative connotations and is associated with social exclusion and stigma (47). A study indicates that an increase in stigma among people living with HIV is associated with a worsening degree of frailty (48).

Emotional reactions are one of the six core elements that make up stigma, and they reflect the negative psychological state of an individual when experiencing illness-related shame. While resilience serves as a core trait of positive psychology (49). Resilience is a protective factor of psychological regulatory capacity. It can help patients cope positively with negative emotions and reduce their psychological stress response (50). Research on Chinese gynecological infertility populations has shown a correlation between higher levels of stigma and lower levels of resilience. In summary, our study hypothesized 3 (H3): stigma mediates the effect between resilience and frailty. In summary, we proposed hypothesis 4 (H4): stigma and health literacy mediate the relationship between resilience and frailty.

**Figure 1:**
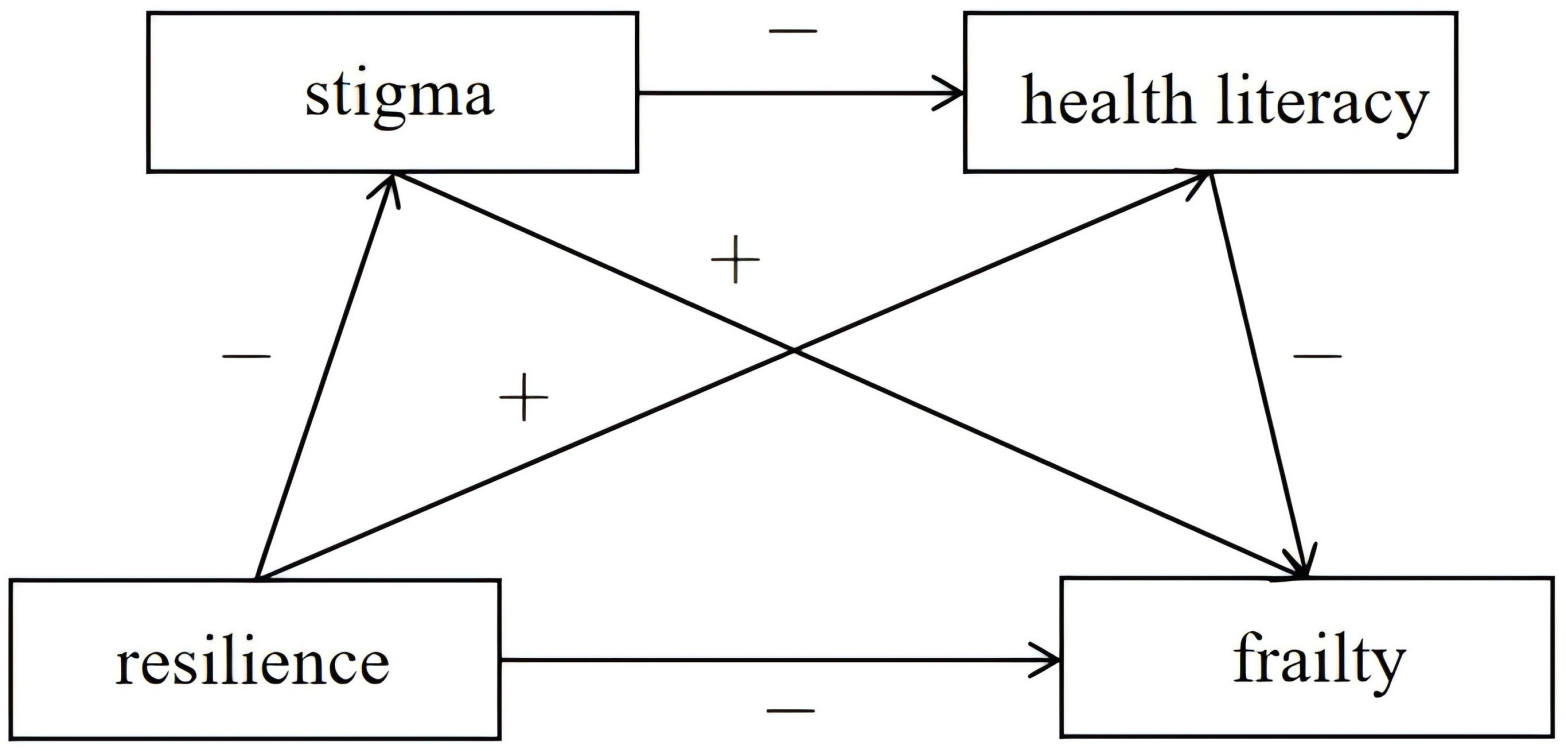
Hypothetical model diagram. 
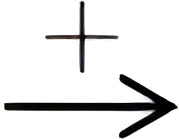
: The former variable positively predicts the latter variable. 
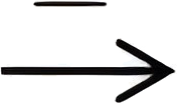
: The former variable negatively predicts the latter variable.

This study intends to explore the effect of resilience on frailty among gynecological patients in oncological treatment and its internal relationship of action, integrating the relationship between resilience, stigma, health literacy, and frailty. Therefore, we made the above four research hypotheses based on the above theoretical and empirical studies. The hypothesized model is shown in **Figure 1**.

## Materials and methods

### Participants and procedure

This was a cross-sectional study conducted between March 2024 and May 2024. A convenience sampling method was used. Among gynecological patients in oncological treatment who met the study criteria were recruited from a tertiary hospital in Wuxi City. Our study used the cross-sectional sample size calculation formula: N=[Z^2α/2^ p(1-p)]/δ^2^, Z_α/2_ is the statistic for the test of significance, Z_α/2_ = 1.96 when α=0.05. p is the prevalence rate, and δ is the tolerance error, generally taken as δ=0.05. Referring to the literature on the prevalence of frailty among gynecological patients in oncological treatment, which ranges from 6% to 60% (8–10), we took p= 10% in the present study. The sample size was calculated N=[1.96^2^×0.1×0.9]/0.05^2^ = 138. Considering the invalid questionnaire, it was decided to increase the sample size appropriately. The final inclusion of 202 cases in this study successfully collected a complete questionnaire that met the quantitative requirements of the study design. Women were selected if they (1) had a clinical diagnosis of gynecologic cancer, (2) were 18 years or older, (3) were undergoing gynecologic oncological treatment in a hospital, and (4) were able to provide written informed consent. Women were excluded if they (1) combined with other malignant tumors, (2) combined with organ failure or life-threatening severe conditions, (3) Patients diagnosed with psychological, mental illness, and language dysfunction.

### Ethical considerations

Before the survey, the researcher explained the study’s nature, purpose, potential risks, and benefits to the participants. All participants were provided written informed consent. The study was approved by the Ethics Committee of the Affiliated Hospital of Jiangnan University (No. LS2023067), which confirmed that all research was conducted in accordance with relevant guidelines/regulations and that informed consent was obtained from all participants and/or their legal guardians. The study was conducted by the Declaration of Helsinki. The researcher followed a standardized process and inclusion-exclusion criteria, and all potentially personally identifiable information was removed from the collected data.

### Measures

#### Demographic characteristics

Demographic characteristics included age, BMI, marital status, education, occupation, per capita monthly income (RMB), primary caregiver, residence, disease types, surgical approach, and frailty.

### Frailty

The Chinese version of the Edmonton Frailty Scale (EFS)was used to measure participants’ frailty. The original version (51) was translated into Chinese by Ge (52). EFS consists of 11 items across nine dimensions: health status, independent living ability, social support, medication, nutrition, emotion, incontinence, cognition, and activity ability. The EFS has a total score of 17 points and is divided into five grades: 0-3 points for no frailty, 4-5 points for vulnerable, 6-7 points for mild frailty, 8-9 points for moderate frailty, ≥10 points for severe frailty. The Chinese version of EFS is reliable and valid and has been used in the Chinese hospitalized population. It was a Cronbach’s alpha coefficient of 0.599 (52).

### Health literacy

The Chinese version of the Health Literacy Management Scale (HeLMS) was used, which was developed by Jordant (53) and translated into Chinese by Sun (54). It consists of 24 items, including four dimensions, namely, information acquisition ability (IAA), communication interaction ability (CIA), health improvement willingness (HIW), and economic support willingness (ESW). Each item was scored on a five-point Likert scale (from 1 to 5). The higher the score, the better the health literacy. The Cronbach’s alpha for the total scale was 0.874, which is reliable and valid, and the Cronbach’s alpha for this scale in this study was 0.882.

### Resilience

Resilience was assessed using the Connor-Davidson Resilience Scale-10 item (CD-RISC-10) (55). It consists of 10 items, all of which were assessed using a 5-point Likert scale (with 1 indicating “never” and 5 indicating “almost always”). The total score ranges from 0-40, the higher the score, the better the resilience. The internal consistency of the CD-RISC-10 scale was good, with a Cronbach’s alpha > 0.85. In the present study, the Cronbach’s alpha was 0.925.

### Gynecologic oncology-related stigma

Gynecologic oncology-related stigma was assessed using the Chinese version of the Social Impact Scale (SIS) (56). It consists of 24 items and includes four subscales: social rejection, financial insecurity, internalized shame, and social isolation. This Likert scale has four response options ranging from strongly disagree (1 point) to strongly agree (4 points). The total inverse scale score for all items ranged from 24 to 96. The higher the stigma score, the higher the level of stigma, with <39 being a low level, 40-59 being a moderate level, and >60 being a high level. The Chinese version of SIS has been shown to have good internal consistency and has been used for multiple populations (57). The Cronbach’s alpha for this scale in this study was 0.860.

### Statistical analysis

SPSS software version 26.0 was used for statistical analysis. Descriptive statistics were performed on the overall data, and categorical data were expressed as frequencies and percentages. The Pearson correlation coefficient was used to analyze the correlation between variables. The relationships between variables were explored using linear regression analysis. Specifically, this analysis was conducted in SPSS using the PROCESS plugin, with Model 6 dedicated to analyzing chained mediation models. i.e., the mediation model was checked using Model 6 in the PROCESS macro of SPSS (58). The significance of the mediation model was verified using bootstrapping 5000 resamples (95% CI). The mediating effect was significant if 95% CI did not include zero. In all analyses, bilateral p < 0.05 was considered statistically significant.

## Results

### Demographic characteristics

A total of 202 gynecological patients in oncological treatment were included. The demographic characteristics of the participants are shown in **Table 1**. The mean age was 49.26 ± 12.56 years, and the mean BMI was 25.03 ± 15.89. The majority of the participants were married (96.5%), had completed high school or higher (50.0%), were employed (72.7%), were currently living in urban areas (67.8%), had a per capita monthly income over 5,000 (60.9%), and had employee medical insurance (72.3%). More than half of the participants had a benign disease (75.7%) and underwent laparoscopic surgery (77.2%). According to the EFS scoring scale, ≥ six was classified as frailty, and 55 (27.2%) had frailty in this study.

**Table 1:** Demographic characteristics of participants (N = 202).

Variable	Category	N (%)
Age (year)	<50	113 (55.9)
	50-59	44 (21.8)
	60-69	29 (14.4)
	>70	16 (7.9)
BMI (kg/m2)	<18.5	5 (2.5)
	18.5-23.9	100 (49.5)
	24.0-27.9	71 (35.1)
	≥28.0	24 (11.9)
Marital status	Single	7 (3.5)
	Married	195 (96.5)
Education	Elementary	45 (22.3)
	Middle school	56 (27.7)
	High school	39 (19.3)
	College	62 (30.7)
Occupation	Unemployed	30 (14.9)
	Worker	13 (6.4)
	Employed	147 (72.7)
	Freelance	9 (4.5)
	Student	3 (1.5)
Per capita monthly income (RMB)	≤1000	4 (2.0)
	1001-3000	16 (7.9)
	3001-5000	59 (29.2)
	>5000	123 (60.9)
Primary caregiver	Self-care	31 (15.4)
	Parents	22 (10.9)
	Children	32 (15.8)
	Spouse	99 (49.0)
	Other	18 (8.9)
Residence	Rural	15 (7.4)
	County	50 (24.8)
	Urban	137 (67.8)
Disease types	Benign	153 (75.7)
	Malignant	49 (24.3)
Surgical approach	Laparoscopy	156 (77.2)
	Abdominal	30 (14.9)
	Hysteroscopy	3 (1.5)
	Other	13 (6.4)
Frailty	No frailty	94 (46.5)
	Vulnerable	53 (26.3)
	Frailty	55 (27.2)
Insurance	Employee medical insurance	146 (72.3)
	Medical insurance for urban and rural residents	47 (23.2)
	Commercial insurance	9 (4.5)

BMI, body mass index.

### Correlation analysis

Correlation analyses between variables are shown in **Table 2**. Resilience was significantly negatively correlated with both stigma (r = -0.545, P < 0.01) as well as frailty (r = -0.395, P < 0.01), and it was positively correlated with health literacy (r = 0.601, P < 0.01). stigma was significantly negatively correlated with health literacy (r = -0.607, P < 0.01) and significantly positively correlated with frailty (r = 0.451, P < 0.01). health literacy was significantly negatively correlated with frailty (r = -0.598, P < 0.01).

**Table 2:** Descriptive statistics and correlation analysis between variables.

Variable	M ± SD	Resilience	Stigma	Health literacy	Frailty
resilience	25.17 ± 5.46	1			
stigma	39.45 ± 8.67	-0.545^**^	1		
health literacy	110.10 ± 9.47	0.601^**^	-0.607^**^	1	
frailty	4.34 ± 2.82	-0.395^**^	0.451^**^	-0.598^**^	1

**p < 0.01.

M, mean; SD, standard deviation.

### The mediating effect of stigma and health literacy between resilience and frailty

As shown in **Table 3**, regression analysis showed that resilience negatively predicted stigma (β = -0.865, p < 0.001); stigma negatively predicted health literacy (β = -0.434, p < 0.001); and resilience positively predicted health literacy (β = 0.666, p < 0.001), and health literacy negatively predicted frailty (β = -0.150, p < 0.001), stigma was not a significant predictor of frailty (β = 0.044, p > 0.05). Resilience was not a significant direct predictor of frailty (β = -0.010, p > 0.05) but was a significant overall negative predictor of frailty (β = -0.204, p < 0.001).

**Table 3:** Regression analysis of variable relationship in model.

Regression equation		Overall fitting index			Significance of regression coefficient		
Outcome variables	Predictor variables	R	R^2^	F	Beta (β)	t	P
Stigma		0.545	0.297	84.535			
	Resilience				-0.865	-9.194	<0.001
Health literacy		0.687	0.472	88.967			
	Resilience				0.666	6.254	<0.001
	Stigma				-0.434	-6.469	<0.001
Frailty	Resilience	0.608	0.370	38.758	-0.010	-0.251	0.802
	Stigma				0.044	1.814	0.071
	Health literacy				-0.150	-6.508	<0.001

R, correlation coefficient; R^2^, coefficient of determination; Beta (β), regression coefficient.

**Figure 2:**
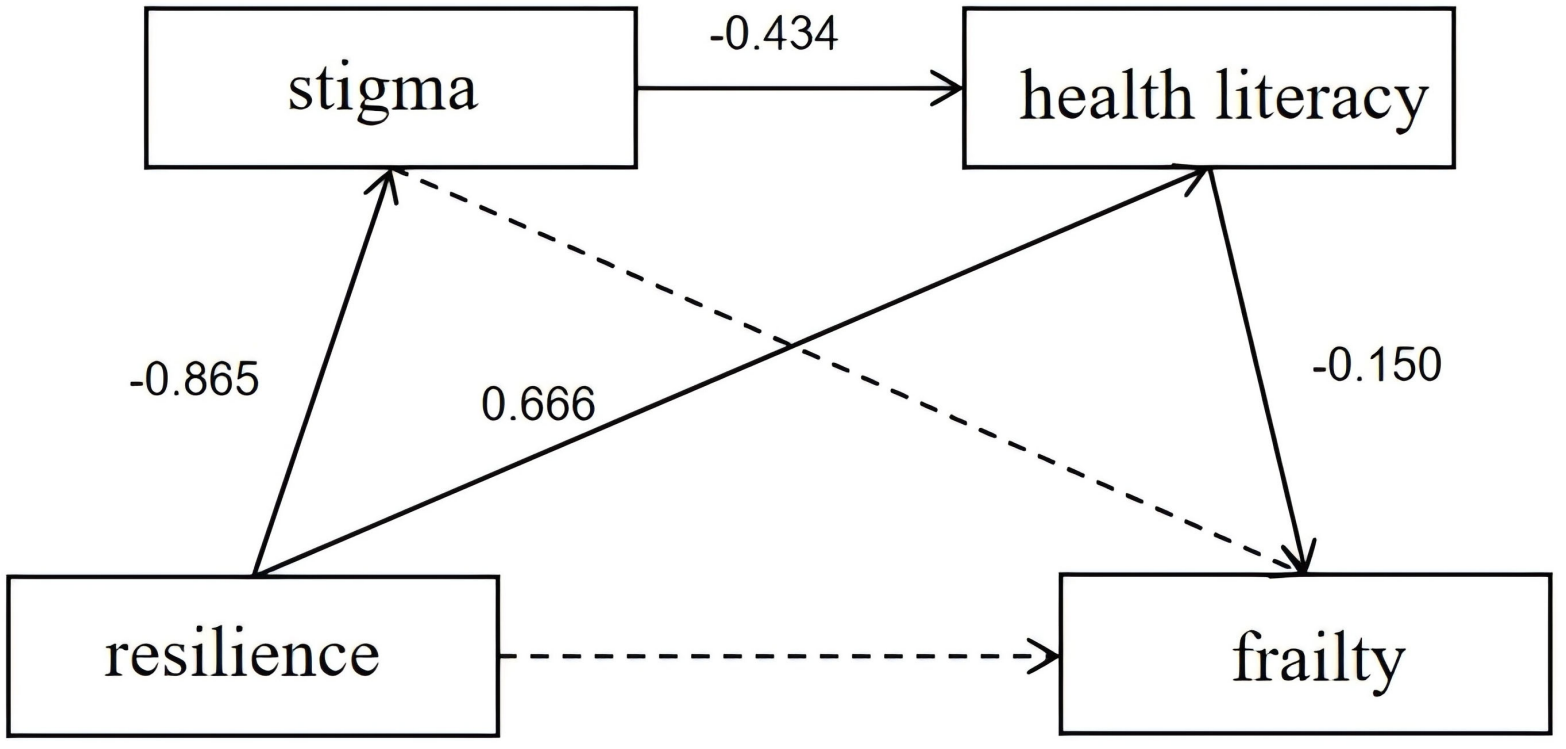
Chain mediation effect diagram. Solid lines: statistically significant predictive effects. Dashed lines: statistically non-significant predictive effects. Numbers: effect values, i.e., the strength and direction of the relationship between the variables.

As shown in **Table 4**; **Figure 2**, the analysis of mediating effects indicated that stigma and health literacy mediated the association between resilience and frailty, with a mediating effect value of -0.194. Specifically, resilience indirectly affected frailty through health literacy (the indirect effect 2 was significant, β = -0.100, 95% CI: -0.155 to -0.053). Additionally, resilience indirectly affected frailty through stigma and health literacy (the indirect effect 3 was significant, β = -0.056, 95% CI: -0.091 to -0.029). However, the independent mediating effect of stigma (indirect effect 1, β = -0.038, 95% CI: -0.085 to 0.005) was insignificant because the 95% CI included 0, indicating that the mediating effect was insignificant. The combined indirect effect of health literacy and stigma-health literacy accounted for 95.10% of the total effect.

**Table 4:** Analysis of the mediating effect of stigma and health literacy.

Effect relation	Beta (β)	LLCI	ULCI	Relative effect size
Indirect effect 1	-0.038	-0.085	0.005	
Indirect effect 2	-0.100	-0.155	-0.053	49.02%
Indirect effect 3	-0.056	-0.091	-0.029	27.45%
Total indirect effect	-0.194	-0.259	-0.133	95.10%
Direct effect	-0.010	-0.084	0.065	
Total effect	-0.204	-0.27	-0.138	

Indirect effect 1: Resilience → Stigma → Frailty.

Indirect effect 2: Resilience → Health literacy → Frailty.

Indirect effect 3: Resilience → Stigma → Health literacy → Frailty.

Beta (β), regression coefficient; LLCI, lower level for confidence interval; ULCI, upper level for confidence interval.

## Discussion

This study used the PROCESS macro program to analyze the relationship between resilience, stigma, health literacy, and frailty in Chinese among gynecological patients in oncological treatment, which helped healthcare professionals develop more precise care strategies.

This study is the first to investigate the frailty among gynecological patients in oncological treatment in China. The results showed that 27.2% of patients experienced frailty during hospitalization. The prevalence of frailty was high (range 6.0% to 60.0%) in patients with gynecologic disorders growing up in Western countries. Differences in the prevalence of frailty may be related to differences in study samples and research tools. Of course, geographic factors and dietary habits may also have a differential impact on frailty.

In the results of this study, resilience was positively correlated with health literacy and negatively correlated with frailty. In addition, health literacy was negatively correlated with frailty. This finding was consistent with previous studies (24, 59, 60). This suggested that health literacy was an important mediator between resilience and frailty(supporting hypothesis 2). This meant that individuals with good resilience and health literacy might be at less risk of developing frailty. Early research reported that resilience can act as an intrinsic force within individuals to help patients develop positive attitudes toward learning and enable them to perform at higher levels of health literacy (61). In addition, another study indicated that resilience as a “ health asset “ is strongly associated with disease regression (62). This can be explained by the fact that patients with high resilience possess greater psychological toughness, which can lead to positive changes in self-illness management attitudes and behaviors and an enhanced sense of disease control, which in turn promotes and maintains overall health. It is also worth noting that the specificity and sensitivity of gynecologic cancers lead to shyness and low health literacy among patients (63). According to the Paasche-Orlow and Wolf Health Literacy Model (27), Patients with low health literacy may not understand how to prevent and control the progression of disease, resulting in poorer overall health. There are programs to improve health literacy for oncology patients, such as tailored rehabilitation programs (TRE) for breast cancer. Thus, healthcare workers can teach about the disease through face-to-face meetings to help patients understand and apply health information; provide resilience skills training through videos (e.g., mindfulness for stress reduction); and encourage patients to participate in treatment decision-making and positive social interactions all of which are measures to improve resilience and health literacy.

In our study, resilience was correlated with stigma and frailty, respectively. This was consistent with previous findings (64–66). Resilience was not a direct predictor of frailty when other factors, such as stigma and health literacy, were also considered. Similarly, stigma was not a direct predictor of frailty. That is, stigma can only play a chain-mediated role through health literacy enhancement(supporting hypothesis 4). In some societies and cultures, receiving treatment for gynecologic oncology is often viewed as a private or sensitive topic. This perception leads women to be reluctant to discuss it openly or seek medical help (67). Previous research suggested that stigma may prevent patients from actively seeking health information, affect their access to and understanding of health knowledge, and exacerbate the negative outcomes associated with low health literacy. People with low health literacy may not realize that feeling shame may have something to do with their abilities (68). Resilience is the process of coordinating internal and external resources for self-regulation, and patients with high resilience actively seek help to mitigate the negative effects of stigma (69). Stigma is a complex social structure product involving multiple interactions and factors (70). These findings suggest that health practitioners should actively promote interactions with patients to reduce stigma by increasing joint patient-provider involvement in the decision-making process while aligning with patient-centered healthcare practice initiatives to encourage patients’ ownership of their health information and management of their health.

Our study found that stigma alone is not a significant mediator, possibly related to several factors. First, stigma is closely linked to the socio-cultural context. It comprises six elements: labeled differences, stereotypes, separation, status loss and discrimination, power, and emotional reaction, all shaped within specific socio-cultural and power structures (37). Secondly, stigma is a dynamic process that changes with the progression of the disease and treatment, and this instability may also contribute to its lack of significance as a mediating variable (71).

The contribution of this study is the clarification of the existence of a chain mediating role of stigma and health literacy between resilience and frailty, which could add to previous literature on the potential mechanisms by which resilience influences frailty. This finding provides new perspectives and theoretical support for clinical healthcare professionals when developing frailty management strategies. Clinical providers should emphasize the implementation of targeted interventions, such as enhancing patients’ health literacy through education and psychological support, as well as reducing stigma, to improve patients’ frailty status. It is recommended that patients’ health literacy be assessed before interventions to better meet their individualized needs and that close attention be paid to patients’ psychospiritual status to reduce stigma. Future studies should further explore how other psychospiritual social factors affect health. Policymakers should consider including stigma in patients’ psychosocial assessment and ensure that the necessary resources and support are available to promote patients’ overall health literacy.

This study has several limitations. First, the sample was selected from a tertiary hospital through a convenience sampling method, which limits the representativeness of the findings to be generalized to the Chinese among gynecological patients in the oncological treatment population. Although we conducted an internal validation cohort, the small size and lack of external validation affected the robustness and generalizability of this study. Future studies should consider using rigorous sampling methods and conducting multicenter studies to enhance sample diversity and reduce selection bias. Second, given that different cancer types and stages may impact outcomes differently, large sample-based subgroup analyses are recommended to reveal the potential impact of these variables. Thirdly, due to the inherent limitations of the cross-sectional design and potential collinearity issues, we can only cautiously interpret the correlations between variables without establishing causal relationships. In the future, we will need to conduct randomized, prospective longitudinal studies. At last, although the prevalence of frailty observed in this study is similar to the existing literature, additional assessment tools are recommended to explore the psychological dimensions of frailty for a more comprehensive understanding of the relationships between variables.

## Data Availability

The original contributions presented in the study are included in the article/**Supplementary Material**. Further inquiries can be directed to the corresponding authors.
