# Supplementary material for: Resilience and frailty among gynecological patients in oncological treatment: the chain mediating role of stigma and health literacy
Source: Front Psychiatry. 2025 Apr 2;16:1497074. doi: 10.3389/fpsyt.2025.1497074 (PMC12010899; doi:10.3389/fpsyt.2025.1497074)
Supplement: Supplementary file 1 [file DataSheet1.pdf]

Health Literacy Management Scale (HeLMS)

	Item	Very Incompetent	Extremely Difficult	Somewhat Difficult	A Little Difficult	No Difficulty
Information Acquisition Ability	Is it challenging to read health brochures?	1	2	3	4	5
	Can you make health decisions based on the information provided by doctors?	1	2	3	4	5
	Do you need help to read written information?	1	2	3	4	5
	Are you able to understand health information encountered in daily life?	1	2	3	4	5
	Are you able to strictly follow medical advice for treatment?	1	2	3	4	5
	Can you go to the hospital alone for medical treatment?	1	2	3	4	5
	Can you obtain the information you need through communication with doctors?	1	2	3	4	5
	Can you fill out medical forms by yourself?	1	2	3	4	5
Communication Interaction	Can you find the information you need from a large amount of information?	1	2	3	4	5
	Are you proactive in seeking answers to things you don't understand about your health?	1	2	3	4	5
	Can you ask family or friends to help you understand health information you don't understand?	1	2	3	4	5
	Do you often participate in healthful activities with fellow patients?	1	2	3	4	5

	Can you prepare for each medical visit?	1	2	3	4	5
	Do you often discuss your health issues with others besides doctors?	1	2	3	4	5
	Can you put the health information you obtain in daily life into practice?	1	2	3	4	5
	Can you ask doctors what you want to know and what you don't understand about treatment measures?	1	2	3	4	5
	If the doctor's explanation is unclear, will you continue asking for clarification?	1	2	3	4	5
	Can you have family or friends accompany you to the doctor?	1	2	3	4	5
Health Improvement Willingness	Are you willing to invest time in health issues?	1	2	3	4	5
	Are you willing to pay attention to your health needs?	1	2	3	4	5
	Are you willing to spend energy to improve your health?	1	2	3	4	5
	Are you willing to improve your health by changing your lifestyle?	1	2	3	4	5
Economic Support	Are you able to afford the cost of medical treatment?	1	2	3	4	5
	Are you able to afford the cost of managing your health?	1	2	3	4	5

Connor-Davidson Resilience Scale （CD-RISC-10）

Item		Never	Rarely	Sometimes	Often	Always
1	I can adapt flexibly when changes occur.	1	2	3	4	5
2	I can handle difficulties.	1	2	3	4	5
3	When facing problems, I respond with humor.	1	2	3	4	5
4	Accumulated experiences make me stronger.	1	2	3	4	5
5	After experiencing illness or hardship, I have a strong ability to recover.	1	2	3	4	5
6	Even when faced with obstacles, I can achieve my goals.	1	2	3	4	5
7	Under pressure, I can concentrate and think about problems.	1	2	3	4	5
8	I do not get upset by failure.	1	2	3	4	5
9	When facing life's challenges, I consider myself a strong person.	1	2	3	4	5
10	I can deal with unpleasant feelings, such as anger.	1	2	3	4	5

Social Impact Scale (SIS)

Item		Strongly Agree	Agree	Disagree	Strongly Disagree
1	The financial difficulties brought by this illness have affected my feelings.	4	3	2	1
2	This disease has affected my work.	4	3	2	1
3	My boss or colleagues will discriminate against me because of my illness.	4	3	2	1
4	The financial difficulties brought by this illness have affected my interpersonal relationships.	4	3	2	1
5	Some people think my abilities could be better.	4	3	2	1
6	Since getting sick, I am not as respected as before.	4	3	2	1
7	I feel that I am not a healthy person anymore.	4	3	2	1
8	I feel that others will worry about catching my illness through contact with me (such as shaking hands with me or eating food I prepared).	4	3	2	1
9	I feel that others avoid me because of my illness.	4	3	2	1
10	I feel that some relatives exclude me because of my illness.	4	3	2	1
11	I think others believe I should blame myself for getting this disease.	4	3	2	1
12	I don't want people around me to know I have this disease.	4	3	2	1
13	I worry that someone will tell others about my disease without my permission.	4	3	2	1
14	I feel that I need to keep my illness a secret.	4	3	2	1

15	I feel that some friends avoid me because of my illness.	4	3	2	1
16	Compared to before, I need to be more certain that others care about me.	4	3	2	1
17	I feel lonelier than before.	4	3	2	1
18	Because of my disease, I feel unequal in my interactions with others.	4	3	2	1
19	I think I am at least partially to blame for getting sick.	4	3	2	1
20	Compared to before, my abilities have declined.	4	3	2	1
21	Because of my illness, I encounter some embarrassing situations.	4	3	2	1
22	Because of my disease, people around me seem awkward and nervous.	4	3	2	1
23	Because of my disease, I sometimes feel useless.	4	3	2	1
24	The changes in my appearance have affected my interactions with others.	4	3	2	1
